# The role of tannic acid and sodium citrate in the synthesis of silver nanoparticles

**DOI:** 10.1007/s11051-017-3973-9

**Published:** 2017-08-04

**Authors:** Katarzyna Ranoszek-Soliwoda, Emilia Tomaszewska, Ewelina Socha, Pawel Krzyczmonik, Anna Ignaczak, Piotr Orlowski, Małgorzata Krzyzowska, Grzegorz Celichowski, Jaroslaw Grobelny

**Affiliations:** 1 Department of Materials Technology and Chemistry, Faculty of Chemistry, University of Lodz, Pomorska 163, 90-236 Lodz, Poland; 2 Department of Inorganic and Analytical Chemistry, Faculty of Chemistry, University of Lodz, Tamka, 91-403 Lodz, Poland; 3 Department of Theoretical and Structural Chemistry, Faculty of Chemistry, University of Lodz, Pomorska 163/165, 90-236 Lodz, Poland; 4 Department of Regenerative Medicine, Military Institute of Hygiene and Epidemiology, Kozielska 4, 01-136 Warsaw, Poland

**Keywords:** Silver nanoparticles, Tannic acid, Sodium citrate, Mixture of tannic acid and sodium citrate, Cyclic voltammetry, Nanoparticle nucleation and growth

## Abstract

We describe herein the significance of a sodium citrate and tannic acid mixture in the synthesis of spherical silver nanoparticles (AgNPs). Monodisperse AgNPs were synthesized via reduction of silver nitrate using a mixture of two chemical agents: sodium citrate and tannic acid. The shape, size and size distribution of silver particles were determined by UV–Vis spectroscopy, dynamic light scattering (DLS) and scanning transmission electron microscopy (STEM). Special attention is given to understanding and experimentally confirming the exact role of the reagents (sodium citrate and tannic acid present in the reaction mixture) in AgNP synthesis. The oxidation and reduction potentials of silver, tannic acid and sodium citrate in their mixtures were determined using cyclic voltammetry. Possible structures of tannic acid and its adducts with citric acid were investigated in aqueous solution by performing computer simulations in conjunction with the semi-empirical PM7 method. The lowest energy structures found from the preliminary conformational search are shown, and the strength of the interaction between the two molecules was calculated. The compounds present on the surface of the AgNPs were identified using FT-IR spectroscopy, and the results are compared with the IR spectrum of tannic acid theoretically calculated using PM6 and PM7 methods. The obtained results clearly indicate that the combined use of sodium citrate and tannic acid produces monodisperse spherical AgNPs, as it allows control of the nucleation, growth and stabilization of the synthesis process.

**Electronic supplementary material:**

The online version of this article (doi:10.1007/s11051-017-3973-9) contains supplementary material, which is available to authorized users.

**Graphical abstract:**
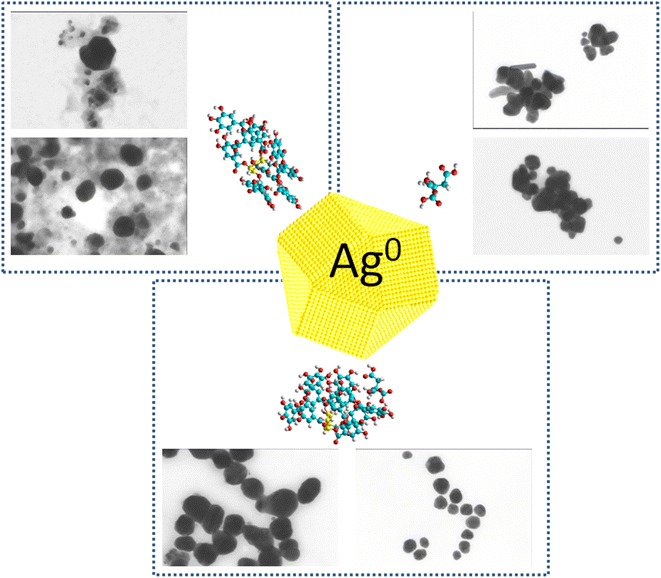
ᅟ

## Introduction

In recent years, the synthesis of nanoparticles (NPs) has been an important topic of research in material science due to the applications of NPs in electronics (Kamyshny et al. 2011), optoelectronics (Tanabe 2007), information storage (Gupta et al. 2012) as well as drug delivery systems and biosensors (Conde et al. 2010; DeLong et al. 2010; Saha et al. 2012; Taton et al. 2000; Tokonami et al. 2012; Yeh et al. 2012). Silver nanoparticles (AgNPs) have found applications in the fields of diagnostics (Larguinho and Baptista 2012; Parveen et al. 2012), therapeutics (Parveen et al. 2012) and catalysis (El-Sayed 2001; Zhang et al. 2008). As in most of these applications, the monodispersity of AgNPs is a desirable feature; hence, synthesis strategies that result in controlled NP size, distribution, shape and stability are still an area of interest.

A variety of methods can be used to synthesize AgNPs: chemical (Dong et al. 2009; Guzman et al. 2009; Kurihara et al. 1995; Panigrahi et al. 2004; Sileikaite et al. 2006; Solomon et al. 2007; Yi et al. 2011), electrochemical (Huang et al. 2006; Johans et al. 2002; Ma et al. 2004), sonochemical (Zhang et al. 2004), thermal decomposition (Kim et al. 2005), photochemical (Henglein 1998), laser irradiation (Abid et al. 2002), microwave irradiation (Yin et al. 2004) and laser ablation (Tsuji et al. 2002), among others. However, uniform, spherical AgNPs can only be obtained using a limited number of methods. Chemical reduction is still the most common strategy for the synthesis of AgNPs, most probably because of the simplicity of the method and apparatus required. In general, preparation of NPs by chemical reduction contains two major steps: reduction (using, for example, borohydrides, sodium citrate, aminoboranes, hydrazine (Tan et al. 2003), formaldehyde, hydroxylamine, saturated and unsaturated alcohols, citric and oxalic acids, polyols, sugars, hydrogen peroxide, carbon monoxide and hydrogen) and stabilization (using, for example, hydrazine (Tan et al. 2003), ascorbic acid (Qin et al. 2010), cetyltrimethylammonium bromide (Khan et al. 2012; Zaheer and Rafiuddin 2012), poly(vinyl alcohol) (Bhatte et al. 2012), poly(vinylpyrrolidone) (Kang and Kang 2011; Zielinska et al. 2009), poly(ethylene glycol) (Luo et al. 2005) sodium citrate (Dong et al. 2009), tannic acid (Yi et al. 2011), dendrimers, polymers and surfactants). Although the preparation of AgNPs by chemical reduction is probably the most versatile method, great care must be exercised to make a stable and reproducible colloid containing spherical NPs.

A typical AgNP chemical synthesis uses either tannic acid or citrate as a reducing and stabilizing agent. Tannic acid contains a central core of glucose that is linked by ester bonds to polygalloyl ester chains. At its natural pH, tannic acid is a weak reducing agent (Tian et al. 2007) that is known to hydrolyse under acidic/basic conditions into glucose and gallic acid units (Bors et al. 2001). It is well known that gallic acid reduces silver nitride at pH > 7 (alkaline) into AgNPs, but the particles form aggregates and agglomerates because gallic acid is a poor stabilizing agent. On the other hand, glucose is known as a weak reducing agent but a good stabilizer at alkaline pH. Sodium citrate can act as both a reducing and a coordinating agent. Free electron pairs in the carbonyl groups can stabilize NPs electrostatically and act as a coordination agent in compounds with metallic atoms that have free orbitals. The main advantage of the chemical reduction method using citrate is the possibility of further NP functionalization (citrate ions can be easily exchanged with other compounds due to the weak interaction of citrate molecules with metal surfaces). Although citrate is most commonly used in AgNP synthesis, it typically results in colloids with a broad size distribution of NPs that exhibit a variety of shapes (Al-Marhaby and Seoudi 2016; Djokic 2008; Dong et al. 2009; El-Kheshen and Gad El-Rab 2012; Gicheva and Yordanov 2013; Lee and Meisel 1982; Pillai and Kamat 2004; Tan et al. 2003). Gicheva and Yordanov used a sodium citrate chemical reduction method and obtained a polydisperse AgNP colloid of polyhedral shapes with individual particle sizes in the 40–100 nm range (Gicheva and Yordanov 2013). Al-Marhaby and Seoudi obtained mostly spherical AgNPs but with an irregular distribution using the reduction of silver nitrate with trisodium citrate dehydrate and sodium borohydride, even with different reagent ratios (Al-Marhaby and Seoudi 2016). Tan and co-workers (Tan et al. 2003) obtained silver nanocrystals of different morphologies using sodium citrate and hydrazine hydrate to reduce silver ions in the presence of aniline. AgNP synthesis using tannic acid also fields polydisperse AgNP colloids with inhomogeneous shapes. Chen and co-workers obtained multipod-shaped Au/Ag nanostructures (Chen et al. 2007) using tannic acid. Cao and co-workers demonstrated that AgNPs with a greater size range (7–66 nm) can be prepared by changing the pH and the molar ratio of tannic acid/AgNO3 (Cao et al. 2014). Zhang and co-workers used tannic acid as both a reducing and a capping agent and synthesized small (8–22 nm) nearly spherical AgNPs for conductive inks (Zhang et al. 2016). Control over the shape and size of AgNPs in chemical reduction is possible with the combined use of citrate and tannic acid in one synthesis (Bastus et al. 2014; Rainville et al. 2013). Dadosh synthesized AgNPs using a single silver reduction step and controlled NP size by varying the concentration of tannic acid in the presence of sodium citrate (Dadosh 2009). As a result, Dadosh obtained AgNPs in the 18–30 nm diameter range with a standard deviation of less than 15%. Bastus and co-workers synthesized monodisperse sodium citrate-coated spherical AgNPs with controlled sizes ranging from 10 to 200 nm via a reduction of silver nitrate using a combination of sodium citrate and tannic acid that allowed the homogenous growth of AgNPs (Bastus et al. 2014). Although the syntheses of AgNP colloids using both sodium citrate and tannic acid are already known, limited effort has been made to understand and experimentally confirm the exact role of these reagents used in combination in the synthesis of NPs.

In this paper, we present the method of synthesis to control the size, size distribution and shape of AgNPs in a chemical reduction method using a combination of sodium citrate and tannic acid. Moreover, in this study, we define and experimentally confirm the exact role of reagents in the synthesis of AgNPs using both reagents. The synthesized AgNPs were characterized using UV–Vis spectroscopy, dynamic light scattering (DLS) and scanning transmission electron microscopy (STEM) techniques. Cyclic voltammetry was used to determine the oxidation and reduction potentials of mixtures of silver, tannic acid and sodium citrate in different molar ratios. The computational modelling approach was used to find possible structures of tannic acid and its adducts with citric acid and to calculate the interaction between them. The compounds present on the surface of AgNPs were identified using FT-IR spectroscopy, and the measured spectrum is compared to that obtained from quantum (PM6 and PM7) calculations. The results confirm that only with the use of two reagents—tannic acid and sodium citrate—is it possible to obtain monodisperse, spherical AgNPs.

## Experimental

### Synthesis of AgNPs

AgNPs were prepared in water by a chemical reduction method. Reagents for the syntheses were of analytical purity and used without further purification: silver nitrate (AgNO_3_, purity 99.999%, Sigma-Aldrich), sodium citrate (C_6_H_5_Na_3_O_7_·2H_2_O, purity 99.0%, Sigma-Aldrich), tannic acid (C_76_H_52_O_46_, Fluka), deionized water (Deionizer Millipore Simplicity system). All solutions of reacting materials were prepared using deionized water. Before use, all glassware was cleaned in a bath of aqua regia solution and rinsed thoroughly using deionized water. The syntheses were carried out using a constant molar ratio of silver nitrate to sodium citrate and tannic acid. For AgNP syntheses with sodium citrate, the molar ratio of silver nitrate to sodium citrate was 1:7; for AgNP syntheses with tannic acid, the ratio of silver nitrate to tannic acid was 1:2; for AgNP syntheses with a mixture of sodium citrate and tannic acid, the ratio of silver nitrate, sodium citrate and tannic acid was 1:7:2. The concentration of silver ions in all solutions was 100 ppm. The syntheses of AgNPs were carried out both at room temperature and at 100 °C. The syntheses at room temperature were carried out by the addition of a sodium citrate and tannic acid mixture to the aqueous solution of silver nitrate. In the case of syntheses at 100 °C, silver nitrate was first heated to its boiling point under reflux and then a mixture of sodium citrate and tannic acid was introduced to the reaction mixture. The solution was heated for additional 15 min and cooled to room temperature. The conditions of syntheses and chemicals used in the preparation of AgNPs are presented in Table 1.

**Table 1:** The conditions of syntheses and chemicals used in the preparation of AgNPs

Sample	Temperature of the synthesis	Reagents		
		Silver nitrate	Sodium citrate (SC)	Tannic acid (TA)
Colloid SC	Room temp.	0.0164%; 95.80 g	4%; 4.20 g	X
Colloid SC*	100 °C			
Colloid TA	Room temp.	0.0158%; 99.35 g	X	5%; 0.63 g
Colloid TA*	100 °C			
Colloid SC–TA	Room temp.	0.0165%; 95.15 g	4%; 4.20 g	5%; 0.63 g
Colloid SC–TA*	100 °C			

X—reagent was not used in the synthesis procedure

#### Precipitates

Directly after synthesis, the colloid was clear, but a brown precipitate appeared at the bottom of the flask (this is not mentioned in other publications) after a few days. To identify the precipitate, FT-IR measurements were performed. Samples were prepared as follows.

- Precipitate 1 (precipitate after the synthesis)

One week after AgNP synthesis, the colloid was decanted from the reaction vessel, leaving behind a precipitate at the bottom. Next, a small amount of deionized water was added to the precipitate and the mixture was centrifuged for 15 min (3600×*g*). A colourless upper layer was collected, and another portion of water was added. Centrifugation was carried out three times following the same procedure. Then, the upper liquid layer was removed and the residual precipitate was analysed by FT-IR spectroscopy.

(b) AgNPs

The AgNP colloid obtained using a mixture of sodium citrate and tannic acid at 100 °C was purified by centrifugation (10,656×*g*) before IR analysis. Centrifugation was repeated five times, and the supernatant was replaced with water. In the last step, the colloid was concentrated. A drop of concentrated colloid was placed on a gold substrate and analysed using IR spectroscopy.

### Measurement techniques

#### UV–Vis spectroscopy

UV–Vis spectra of silver colloids were recorded using light source versatile lamps optimized for the visible-near infrared (vis-NIR) (360–2000 nm) (Ocean Optics, HL-2000, tungsten halogen light sources, USB2000 with detector (miniature fibre optic spectrometer)).The presented results were obtained by averaging 1000 single measurements.

#### Dynamic light scattering

The size and size distribution of AgNPs in colloids were measured using DLS (Nano ZS zetasizer system; Malvern Instruments; laser wavelength of 633 nm (He–Ne); scattering angle 173°; measurement temperature 25 °C, medium viscosity 0.8872 mPa s; medium refractive index 1.330). Before taking the DLS measurement, the colloid was filtered (0.2 μm polyvinylidene fluoride (PVDF) membrane). All measurements were performed in a quartz microcuvette. Five measurements were taken, and the mean result was recorded.

#### Scanning transmission electron microscopy

The size and shape of AgNPs were determined using high-resolution STEM (FEI, NovaNanoSEM 450; accelerating voltage 30 kV). The AgNPs samples for STEM measurements were prepared by deposition of the colloid to carbon-coated copper grids. A size histogram was made after measuring the size of at least 100 NPs.

#### Cyclic voltammetry

Electrochemical measurements were carried out using CV. The measuring equipment consisted of a PAR 273A potentiostat (EG&G Princeton Applied Research Company) connected to a computer with the CorrWare 2.9 and CorrView 2.9 software (Scribner Associates, Inc.). All electrochemical measurements were carried out in a conventional three-electrode cell where a gold electrode (with an area of 0.96 cm^2^) was used as the working electrode, a platinum sheet as the counter electrode and a saturated calomel electrode (SCE) in NaCl as the reference electrode. The sweep potential rate (*v*) was 100 mV s^−1^.

#### FT-IR spectroscopy

A Nicolet iS50 FT-IR spectrometer with an MCT detector and a grazing angle attenuated total reflectance (GATR) grazing angle attenuated total reflectance (ATR) accessory (the GATR features a 65° incident angle and a hemispherical germanium ATR crystal) were used to examine the precipitates (resolution of 4 cm^−1^). A sample was placed dropwise on a gold substrate, and following solvent evaporation, it was put on an ATR crystal, and measurements were taken.

## Results

**Fig. 1:**
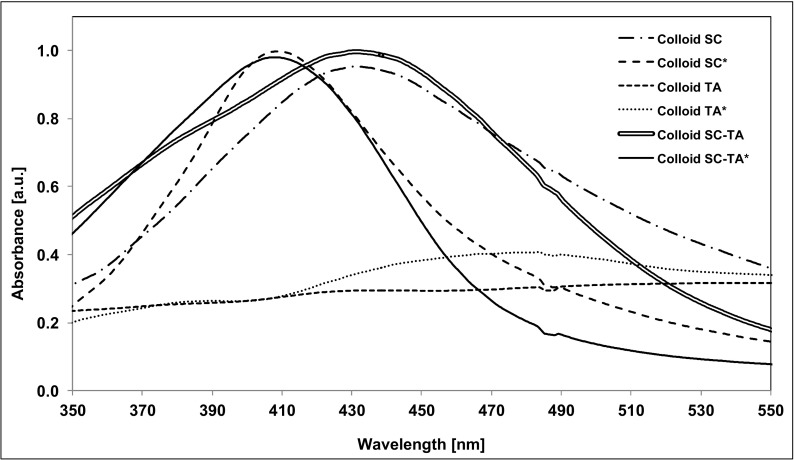
UV–Vis spectra of AgNPs synthesized using sodium citrate (Colloid SC), (Colloid SC*); tannic acid (Colloid TA), (Colloid TA*); and a mixture of sodium citrate and tannic acid (Colloid SC–TA), (Colloid SC–TA*) at room temperature (Colloid SC, TA, SC–TA) and heated to 100 °C (Colloid SC*, TA*, SC–TA*)

UV–Vis spectroscopy is a sensitive method for detecting colloidal silver, because AgNPs exhibit a characteristic absorption peak at about 400 nm, which is attributed to surface plasmon excitation. Metal NPs have free electrons, which give the surface plasmon resonance (SPR) absorption band due to the combined vibration of electrons of metal NPs in resonance with the light wave. Figure 1 shows the UV–Vis absorption spectra of AgNPs synthesized using sodium citrate (SC), tannic acid (TA) and a mixture of both sodium citrate and tannic acid (SC–TA) at room temperature and at 100 °C.

The absorption band maximum for Colloid SC and Colloid SC–TA was observed at *λ* = 430 nm and *λ* = 432 nm, respectively. For the same syntheses but carried out at 100 °C (Colloid SC* and Colloid SC–TA*), the maximum of the absorption band is shifted to shorter wavelengths and equals *λ* = 408 nm, which is characteristic of smaller NPs. No characteristic peak was observed in colloids synthesized using tannic acid (Colloid TA and Colloid TA*).

**Fig. 2:**
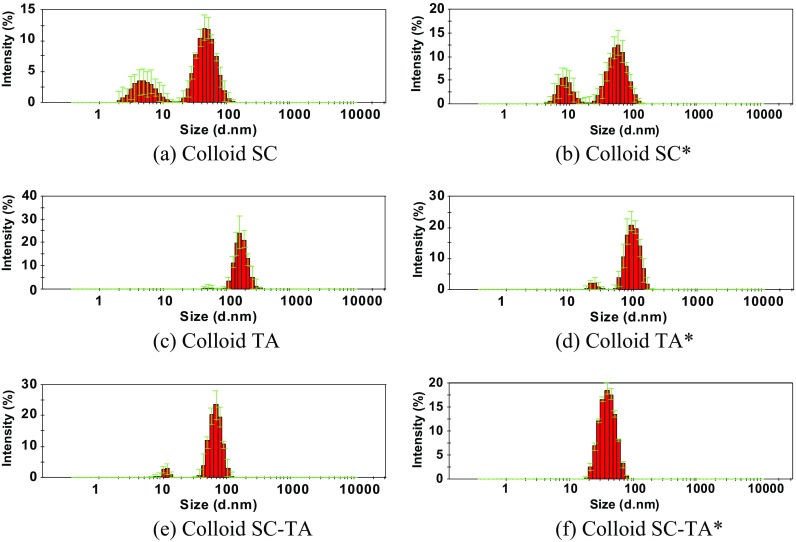
DLS size distribution histograms of AgNPs synthesized using sodium citrate (**a**, **b**), tannic acid (**c**, **d**) and a mixture of sodium citrate and tannic acid (**e**, **f**) at room temperature (**a**, **c**, **e**) and heated to 100 °C (**b**, **d**, **f**)

The colloidal state and the hydrodynamic size of AgNPs in the case of all samples were investigated using DLS (Fig. 2).

**Fig. 3:**
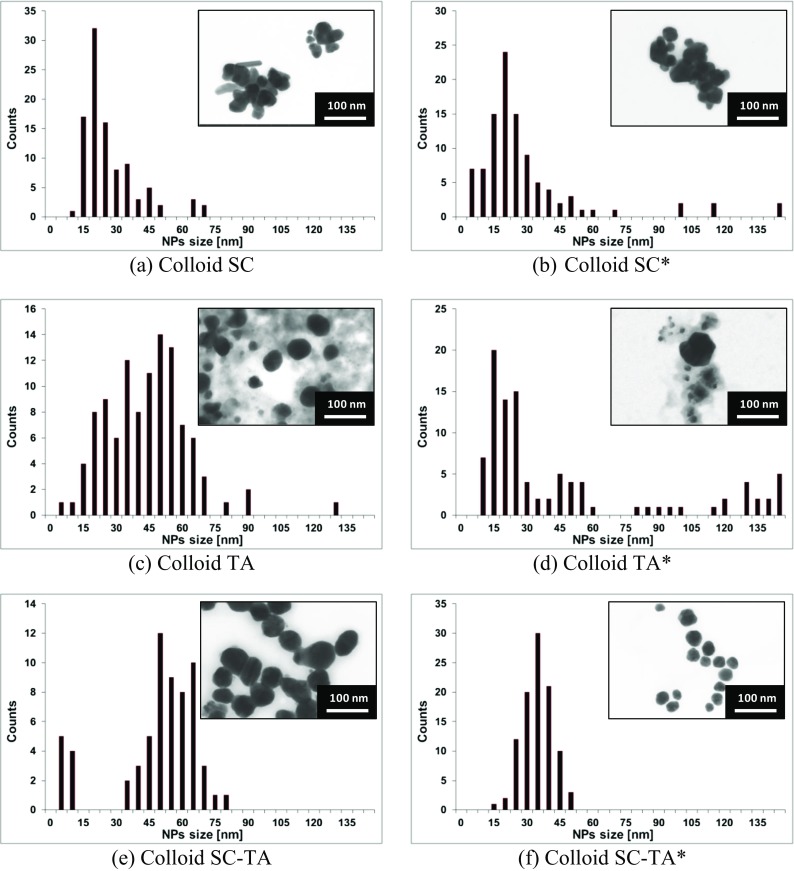
STEM images and size distribution histograms of AgNPs synthesized using sodium citrate (**a**, **b**), tannic acid (**c**, **d**) and a mixture of sodium citrate and tannic acid (**e**, **f**) at room temperature (**a**, **c**, **e**) and heated to 100 °C (**b**, **d**, **f**)

The size and size distribution of synthesized AgNPs were determined using STEM (Fig. 3). The particle size histogram was obtained by measuring at least 100 NPs.

AgNPs with different sizes and a diversity of shapes were obtained in syntheses using sodium citrate. In syntheses using tannic acid, mostly spherical particles in a large variety of sizes (5–140 nm) were obtained. AgNPs of homogenous shape and size were obtained in syntheses carried out using a mixture of citrate and tannic acid (Colloids SC–TA and SC–TA*). A summary of AgNP colloid characterization is shown in Table 2.

**Table 2:** The overall results obtained for the syntheses of AgNPs using sodium citrate (SC) tannic acid (TA) and a mixture of sodium citrate and tannic acid (SC–TA) at room temperature (Colloid SC, TA, SC–TA) and heated to 100 °C (Colloid SC*, TA*, SC–TA*)

Reagent	Sample	*λ*_max_ (nm)	DLS size (nm)	PdI	STEM size (nm)
Sodium citrate	Colloid SC	430	5 ± 2; 50 ± 18	0.614	15–50
	Colloid SC*	408	9 ± 2; 58 ± 20	0.538	5–45; >100
Tannic acid	Colloid TA	–	170 ± 27	0.114	15–65
	Colloid TA*	470	24 ± 3; 99 ± 26	0.189	5–55; >100
Sodium citrate/tannic acid mixture	Colloid SC–TA	432	11 ± 1; 68 ± 15	0.228	46 ± 19
	Colloid SC–TA*	408	40 ± 12	0.168	32 ± 6

The use of a single reagent for the synthesis of AgNPs (either sodium citrate or tannic acid) results in particles with a broad range of sizes and geometries. AgNPs synthesized with sodium citrate alone are inhomogeneous in both size and shape. The sole use of citrate (a weak reducing agent and a strong complexing stabilizer) does not allow the control of AgNP morphology. Similarly, AgNP syntheses using just tannic acid lead to polydisperse colloids. Even a change in reaction conditions, that is, the increase of the synthesis temperature to 100 °C, does not produce uniform AgNPs. The reduction of a silver salt using a mixture of sodium citrate and tannic acid at 100 °C gives particles of ~30 nm diameter with a narrow size distribution. The combined use of sodium citrate and tannic acid enables control over the nucleation, growth and stabilization processes, which leads to reproducible monodisperse AgNPs. The overall results indicate that the presence of both tannic acid and sodium citrate in the synthesis of AgNPs, as well as a reaction temperature of about 100 °C, promote the homogenous growth of particles.

The obtained data indicate that a sodium citrate and tannic acid mixture has better reducing and stabilizing properties in AgNP synthesis than either sodium citrate or tannic acid alone. This may suggest that the formation of a complex of citrate and tannic acid is involved in NP synthesis. To confirm this hypothesis, we performed voltammetric measurements.

**Fig. 4:**
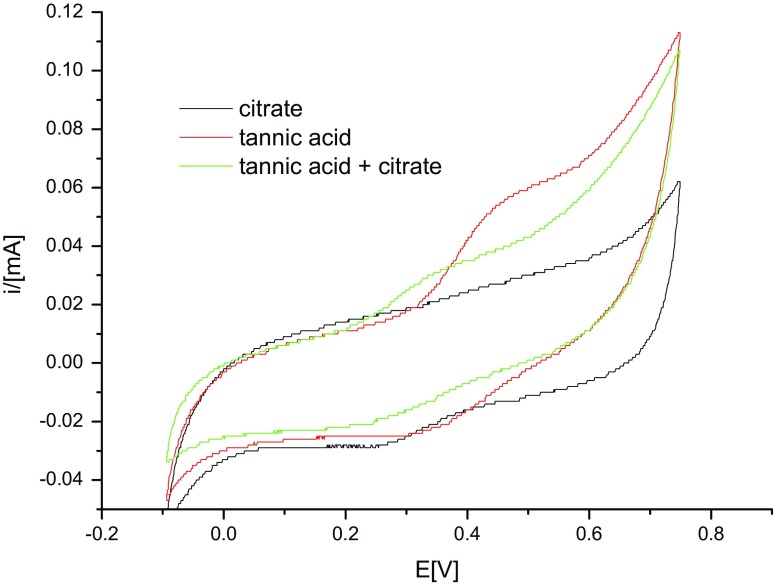
The voltammetric curves obtained for solutions of sodium citrate (*black*), tannic acid (*red*), sodium citrate and tannic acid (*green*) (colour figure online)

Voltammetric measurements were taken to determine the potentials of reduction and oxidation and the changes of these potentials under the influence of substances in the solution. At the same time, the pH of all solutions was measured. In the first stage of electrochemical research, voltammetric measurements were carried out on a solution of sodium citrate (*c* = 10^−3^ M), tannic acid (*c* = 10^−3^ M) and a solution containing both sodium citrate and tannic acid (*c* = 10^−3^ M each). All the solutions contained sodium nitrate as a basic electrolyte (*c* = 10^−3^ M). The obtained voltammetric curves are presented in Fig. 4.

Tannic acid in the solution containing sodium citrate undergoes oxidation the most readily. Oxidation starts at a potential of 0.195 V. This value is 0.115 V smaller than in the case of the solution containing tannic acid alone. The reason for such a change in the potential might be the interaction between the particles of tannic acid and citrate anions. Probably, these are interactions of the type of hydrogen bonds between the hydroxyl groups of tannic acid particles and the oxygen atoms of carbonyl groups of citrates. The formation of stable citric acid (CA)–TA complexes was also confirmed by quantum calculations (Table 3 and Fig. 8).

**Table 3:** The interaction enthalpies in the CA–TA and CA–TA_ox_ adducts shown in Fig. 8

Adduct	Interaction enthalpy (kcal mol^−1^)
CA–TA_Gin_	−27.43
CA–TA_Gex_	−20.75
CA–TA_oxGin_	−22.49
CA–TA_oxGex_	−17.53

Calculations performed assuming that only one gallic group is oxidized. ex, in—external, internal—the position of the gallic acid group which is assumed to interact directly with CA at the beginning of each simulation

**Fig. 5:**
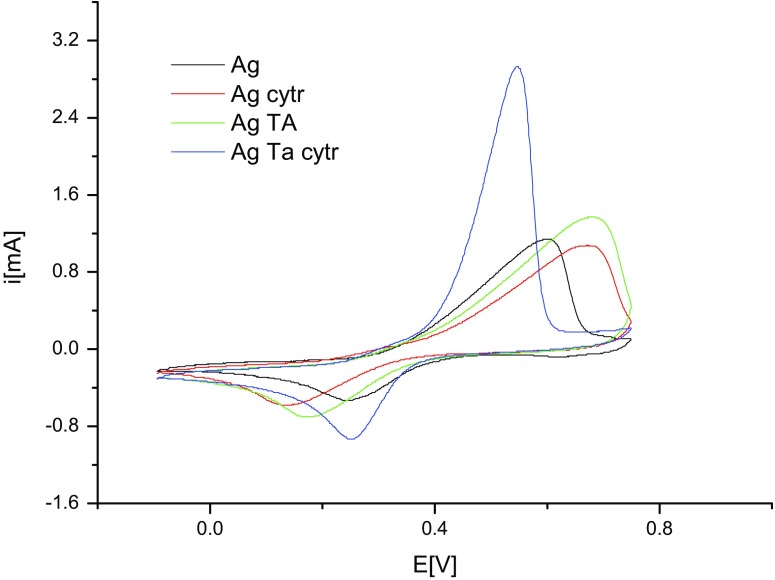
The voltammetric curves obtained for solutions of silver ions (*black*), silver ions with sodium citrate (*red*), silver ions with tannic acid (*green*) and silver ions with sodium citrate and tannic acid (*blue*) (colour figure online)

The next stage of the research was to carry out voltammetric measurements of solutions containing silvers ions. Measurements were carried out on the following aqueous solutions: silver ions, silver ions and sodium citrate, silver ions and tannic acid and silver ions and both sodium citrate and tannic acid. The concentration of each substance was *c* = 10^−3^ M. All the solutions contained sodium nitrate as a basic electrolyte (*c* = 10^−3^ M). As seen in the voltammetric curves presented in Fig. 5, the peak potential of the reduction of silver ions in a basic electrolyte is 0.244 V.

**Fig. 6:**
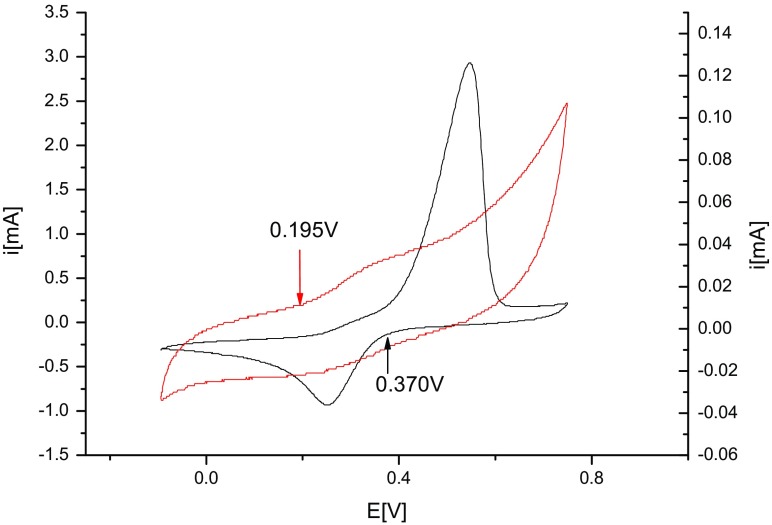
The voltammetric curves obtained for solutions of silver ions with sodium citrate and tannic acid (*black*) and sodium citrate and tannic acid (*red*) (colour figure online)

In the solution containing silver ions and sodium citrate, the peak potential is shifted to 0.134 V. The reduction of silver ions in this case is difficult, which is due to the complexation of silver ions by the citrate. The formation of silver complexes with citrates has already been described in the literature (Dadosh 2009). In the solution containing silver ions and tannic acid, the peak potential of the reduction of silver is0.173 V. In this case, the reduction of silver is also difficult. The probable cause is the complexing of the silver ions by tannic acid. Similar interactions of tannic acid with other metals have already been described (Zhang et al. 2004, 2016). In the case of the solution containing silver ions, sodium citrate and tannic acid, the peak potential of the reduction of silver is 0.252 V. Moreover, the peak current in this case is much higher than for other solutions. The value of the reduction indicates that the reduction in this solution proceeds as easily as in the solution containing only basic electrolyte and that it proceeds twice as fast, which is suggested by the value of the current. A comparison of the potential of the oxidation of tannic acid (0.195 V) and the potential of the reduction of the silver ions (Fig. 6) in solutions containing sodium citrate and tannic acid shows that the reduction of silver should proceed spontaneously at room temperature. This conclusion is in accordance with what was observed in the reactions.

The voltammetric measurements revealed that the reduction potential of the CA–TA mixture is lowered compared to the potentials of CA and TA separately. This indicates that the complex acts as a reducing agent in the synthesis of AgNPs. In the case of syntheses carried out at room temperature, the rate of reaction is slower compared to syntheses carried out at 100 °C; hence, the size of the obtained AgNPs is larger and more polydisperse (Table 2).

To investigate the possibility of the formation of stable complexes of CA with tannic acid (TA) in aqueous solution, we applied a theoretical approach involving computer simulations and the semi-empirical PM7 method. Considering how large and complex the TA and CA–TA structures are, the configurational space of each is vast and its exhaustive exploration would require extensive computations. In this work, an attempt to find the lowest energy geometries in solution was made by performing relatively short conformational searches, thus giving only a preliminary insight into the structural and energetic properties of these systems. A detailed description of the calculation method is given in Supplementary material.

**Fig. 7:**
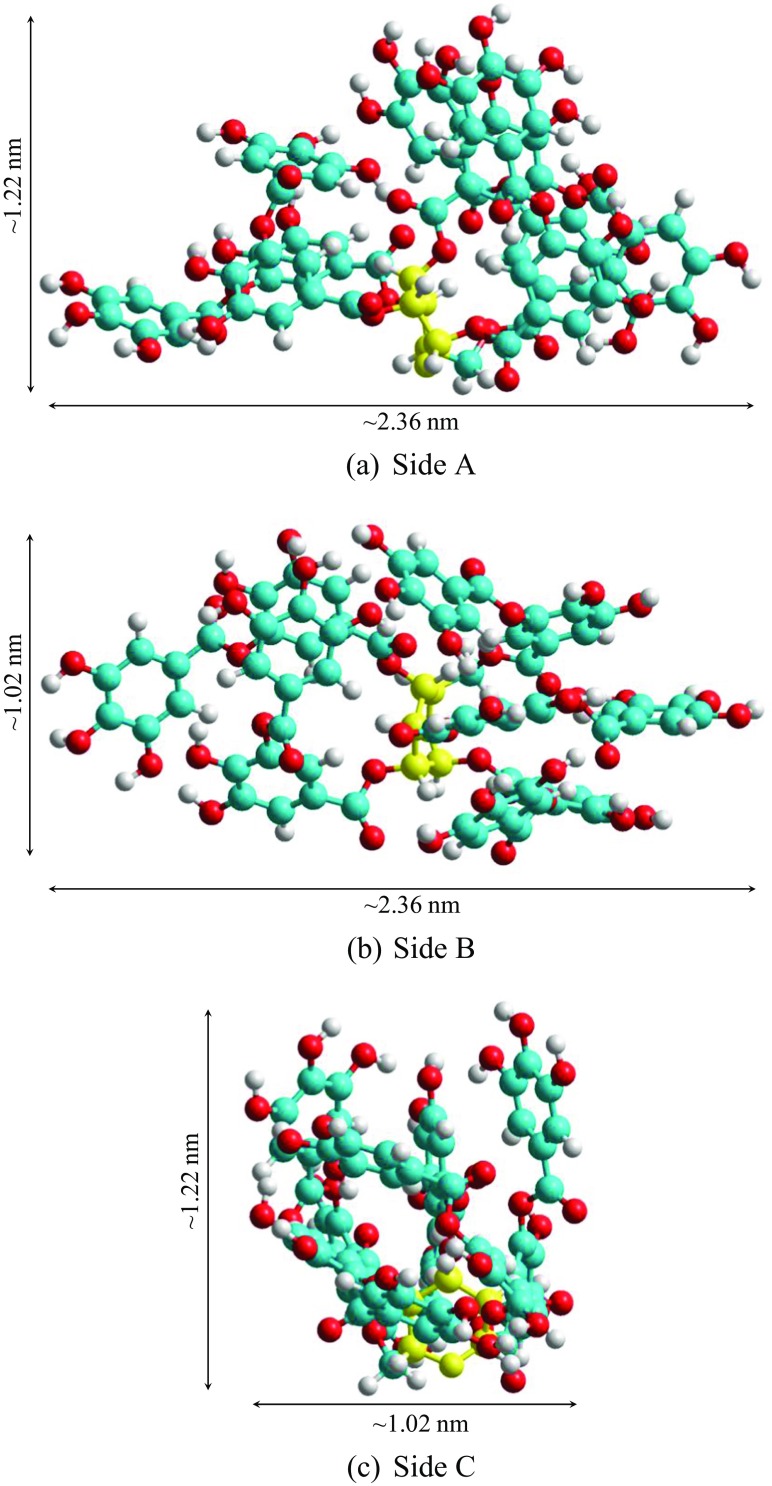
The lowest energy structure of TA (a view is given from three different sides with corresponding dimensions) found from the simulations and used to create adducts with citric acid (*yellow* indicates the glucose ring; *blue* are carbons in gallic units; *red* represents oxygen atoms and *grey* indicates hydrogen atoms) (colour figure online)

First, the structure of tannic acid in water was studied. In Fig. 7, we present the lowest energy structure of TA (shown from three different sides with the approximate dimensions) found from simulations. This structure was used to construct the CA–TA adducts in a further study of their structures and the strength of interaction between TA and CA. However, it must be understood that this structure is just one of the many various conformers that can be present in the solutions.

Considering the steric hindrance in the tannic acid structure, one could expect that only external gallic units (those most distant from glucose) may be involved in the oxidation reaction. However, the structure presented in Fig. 7 shows that such an assumption may be incorrect. The π electrons of aromatic rings belonging to different gallic units interact, and the ‘branches’ are arranged into groups containing three and two parallel elements (Fig. 7b, c). Hence, the internal gallic units (directly bound to glucose) are exposed and can also be involved in the complexation reaction.

**Fig. 8:**
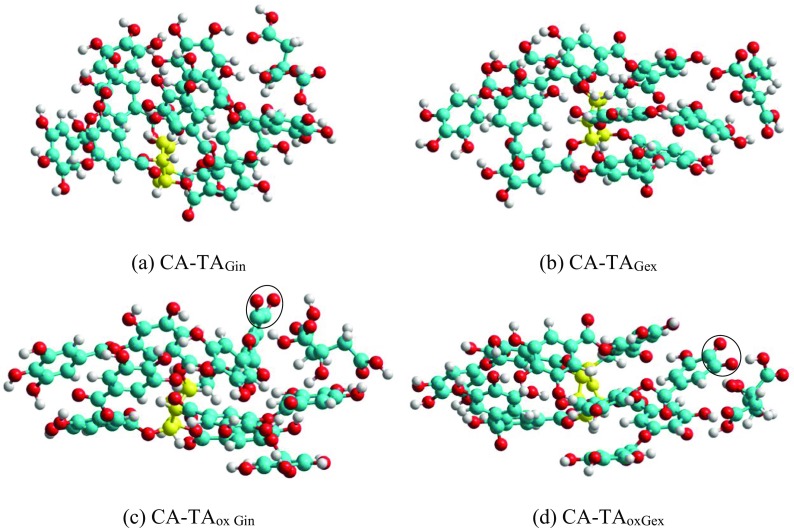
The lowest energy structures found from the simulations of the adducts of citric acid (CA) with tannic acid (TA) or its oxidized form (TA_ox_). The notation ‘in’ and ‘ex’ refer to the initial position of CA in the simulation. **a** CA–TA_Gin_—CA near the internal gallic unit of TA. **b** CA–TA_Gex_—CA near the external gallic unit of TA. **c** CA–TA_ox Gin_—CA near quinone of TA_ox_ (corresponding to the internal gallic unit in TA). **d** CA–TA_oxGex_—CA near the quinone of TA_ox_ (corresponding to the external gallic unit in TA). *Black circles* in (**c**, **d**) indicate the position of quinine C=O groups

In the next step, a series of simulations was performed to evaluate the strength of interactions between TA and CA. Both TA and TA with one oxidized gallic unit (to quinone) that may be formed during AgNP synthesis were included in the analysis. The intermolecular interactions of CA with either internal or external gallic units of TA were evaluated by placing CA near the unit of interest and performing the simulation to find its energetically favoured orientation. The enthalpies of interaction shown in Table 3 were calculated for the most stable structures obtained from the simulations (Fig. 8). Our preliminary results predict that the intermolecular interactions between CA and TA and TA_ox_ are of similar magnitude, but in the CA–TA complex, it is several kcal/mol stronger than in CA–TA_ox_. The results also suggest that the interactions in the structures ‘ex’ shown in Fig. 8b, d are weaker than in the structures ‘in’ (Fig. 8a, c).

The structures of possible CA–TA adducts with the lowest energies formed in the aqueous solution are shown in Fig. 8. The size of the CA–TA adducts is comparable with the tannic acid molecules (i.e. maximum 1.2 × 2.4 nm).

Considering the colloid composition, tannic acid, citrate as well as adducts of TA–CA can be present on the surface of NPs. However, taking into account the DLS results (hydrodynamic size of AgNPs containing the metallic core of NPs and the hydration coating on NPs), compounds of larger sizes are present on the surface of NPs (see Table 1). The sizes of CA (0.86 × 0.40 × 0.35 nm), simulated TA (1.22 × 2.36 × 1.02 nm) structures and CA–TA complexes (~2.4 nm) indicate that TA molecules and adducts of CA–TA are adsorbed on AgNPs over citrate ions. However, identifying which adducts are actually present on the surface of AgNPs following synthesis is not trivial and requires further investigation.

**Fig. 9:**
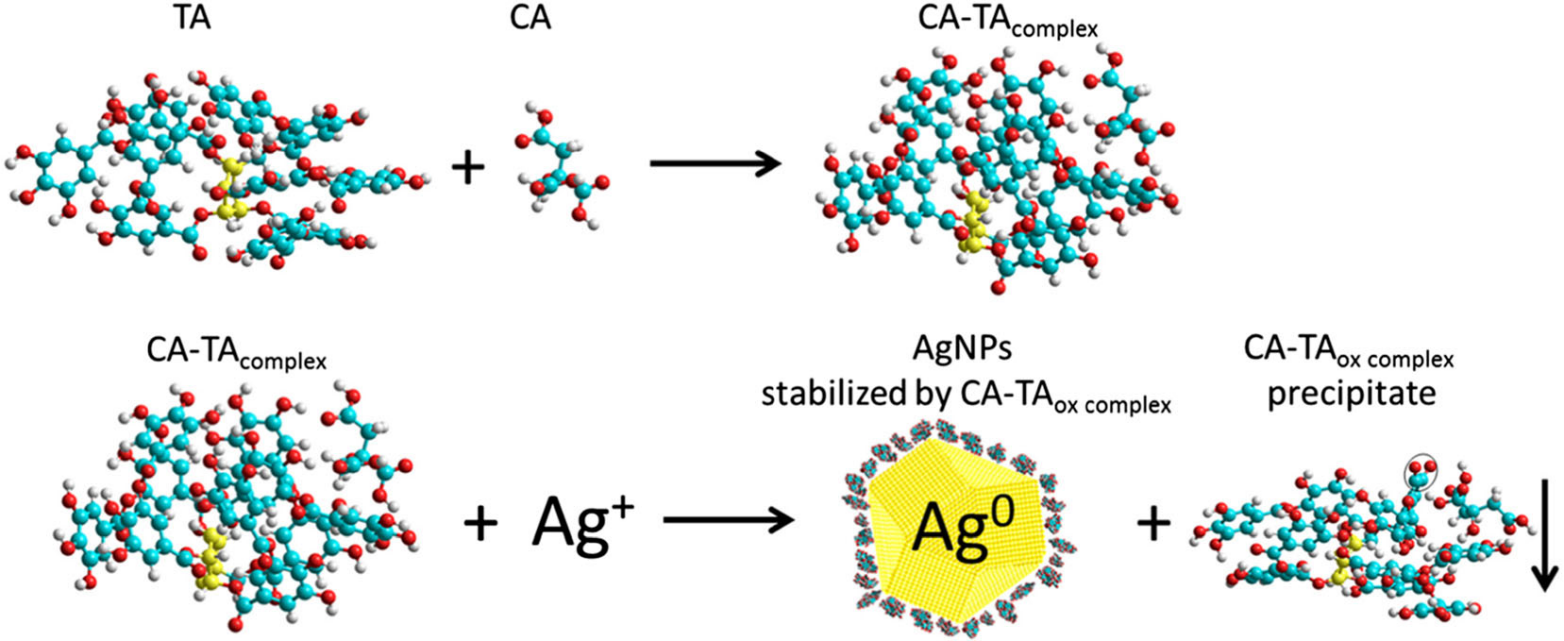
Scheme of reduction of silver ions by a sodium citrate–tannic acid complex

On the basis of the results of our voltammetric measurements and computer simulations, we propose a scheme of silver ion reduction by a sodium citrate–tannic acid complex (Fig. 9). In the first stage of the reaction, the CA–TA complex is formed. The adduct shows better reducing properties than either CA or TA alone. In the next stage, the CA–TA complex reduces silver ions to metallic Ag^0^, which is then stabilized with CA–TA_Gin_, CA–TA_Gex_, CA–TA_ox Gin_ and CA–TA_oxGex_ present in the colloid solution.

The CA–TA complex is being formed before AgNP formation starts (the mixture of CA and TA is prepared a few minutes before the reaction in a separate vessel, see Synthesis of AgNPs). Hence, the rate of complex formation should not influence the final formation of AgNPs. Moreover, free CA and TA molecules not included in complexes have a negligible effect on the obtained AgNPs because of their weaker reducing properties (voltammetric measurements). The formation of CA–TA adducts as well as the oxidation of gallic units in tannic acid during the reduction of Ag^+^ may result in lowering the solubility of the complex and result in precipitate formation (which is not mentioned in the literature). The precipitate appears not only in the AgNP colloid but also in the CA–TA mixture (after about a week of storage). Precipitate formation results from the reduction reaction in the case of the AgNP colloid and from the partial oxidation of TA (which is involved in the complex) with the oxygen in the air in the case of the CA–TA mixture. The storage of TA does not result in precipitation; hence, precipitate formation must be closely associated with the CA–TA complex. To confirm the presence of partially oxidized TA in the complex and to identify the compounds adsorbed on the surface of AgNPs, FT-IR measurements were examined.

**Fig. 10:**
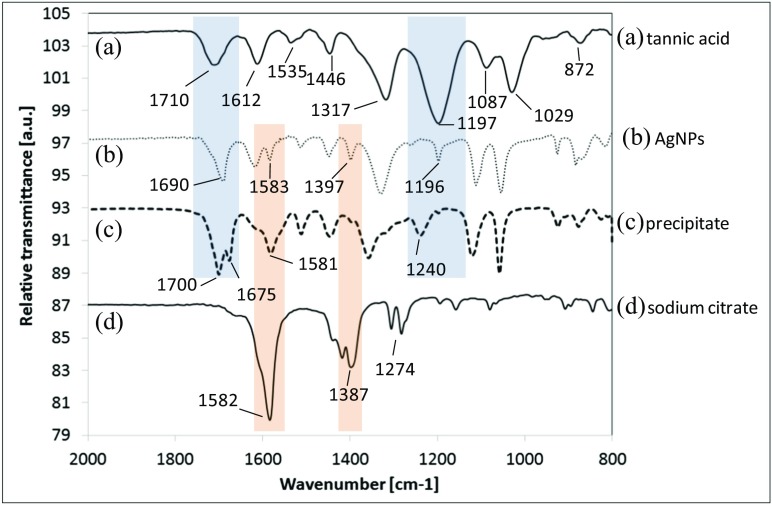
IR spectrum of (*a*) tannic acid, (*b*) AgNPs, (*c*) precipitate after the AgNP synthesis and (*d*) sodium citrate

IR spectroscopy was performed on the evaporated purified AgNPs and purified sedimented precipitate after AgNP synthesis, and the results are compared with the spectra of sodium citrate and tannic acid (Fig. 10). There are many IR spectra of tannic acid in the literature (Cowen and Al-Abadleh 2009; Falcao et al. 2014). Tannic acid is a substance extracted from plants; hence, the IR spectrum of this natural compound (band intensity and/or its position) may be slightly different depending on the origin of the tannic acid. To correctly identify the bands characteristic of tannic acid, we compared the measured spectrum of tannic acid with the computed spectrum obtained from the vibrational analysis performed using the PM7 and PM6 methods (see Fig. 1S and Table 1S in Supplementary material).

All IR spectra were obtained using the GATR accessory. This method allows us to obtain a more intense signal at lower wavelengths compared to standard transmission spectra obtained using KBr. The analysis of the IR spectrum of TA and CA is crucial in describing processes taking place during AgNP synthesis. The characteristic bands for TA were observed at 1710 (C=O stretching), 1612 (C_ar_–C_ar_ stretching), 1317 (C_ar_–OC stretching, C_ar_–O–H in-plane bending, C_ar_–C_ar_ stretching), 1197 cm^−1^ (O–CO and C_ar_–CO stretching, C_ar_–O–H in-plane bending) and for sodium citrate at 1582 cm^−1^ (C=O stretching asymmetric in COO–), 1387 cm^−1^ (C–OH stretching), 1274 cm^−1^ (C–H stretching deformation). The analysis of the characteristic regions marked in colour in Fig. 10 (blue and red areas) allows us to identify the compounds present on AgNPs as well as to determine the composition of the precipitate sedimented after the AgNP synthesis. A band characteristic of quinone units in the region 1750–1650 cm^−1^ (C_ar_=O stretching) was observed in the spectrum of both precipitate (two maxima at 1700 and 1675 cm^−1^,C_ar_ = O stretching in *ortho* and *para* positions) and AgNPs (1690 cm^−1^). This indicates that tannic acid was oxidized to quinine (Socrates 2001) during the synthesis of metal NPs and confirmed the role of TA as a reducing agent in the reaction. The intensity of the band characteristic of O–H (at 1240 cm^−1^) is smaller in the precipitate compared to the band for TA which indicates that OH groups are oxidized to C=O in the precipitate, which is further proof of the oxidation of TA. This indicates that a precipitate contains oxidized forms of tannic acid (quinone units). The precipitate spectrum is significantly changed compared to TA and also contains the band characteristic of citrate at 1581 cm^−1^. This proves that the precipitate consists of sodium citrate–tannic acid complexes, the structures of which were simulated and are shown in Fig. 8c, d. The spectrum of AgNPs contains bands characteristic of both TA (1690 and 1196 cm^−1^) and citrate (1583 and 1397 cm^−1^). However, the bands’ position is shifted compared to the precipitate, which may be because the spectrum was collected from the surface of AgNPs. The intensity of the OH band versus C=O band changed proportionally as in the case of the precipitate. This indicates that the part of the OH groups was oxidized to quinone. However, the band from the quinones is shifted from 1700 cm^−1^ observed in the precipitate to 1690 cm^−1^ in the case of AgNPs. In the spectrum of AgNPs, the bands characteristic of citrate are also present at 1583 cm^−1^ (C=O stretching asymmetric in COO–) and a weak signal at 1397 cm^−1^ (C=O stretching symmetric in COO–) was also observed. The intensity of these bands is higher than those present in the precipitate. This indicates that on the surface of AgNPs, it is likely that sodium citrate–tannic acid complexes are present as well as citrate and tannic acid, while the precipitate contains mostly complexes of sodium citrate–tannic acid (see Fig. 8c, d).

## Conclusions

We herein reported a method for the synthesis of monodisperse AgNPs based on the reduction of silver nitrate by a combination of two agents, sodium citrate and tannic acid, via chemical reduction. The use of a mixture of tannic acid and sodium citrate allowed the preparation of NPs homogenous in size (about 30 nm) and shape. The role of tannic acid and sodium citrate was determined using FT-IR, voltammetric measurements and computer simulations. Both voltammetric measurements and modelling of conformation space confirm the formation of a CA–TA complex, which acts as a reducing agent in the synthesis of AgNPs. The IR spectral analysis indicated that tannic acid (a part of the CA–TA complex) was oxidized to quinone in the synthesis of AgNPs, which confirms the role of CA–TA as a reducing agent in the reaction. As a result, metallic silver is formed in NPs stabilized by the CA–TA_ox_ complex while free complex molecules (unadsorbed on AgNPs) form a precipitate in a colloid solution after a week of a storage.

The presented results give a deeper understanding of the role of tannic acid and sodium citrate in the synthesis of AgNPs. Only the combined use of citrate and tannic acid produces the active CA–TA complex that enables us to precisely control the reaction conditions for the preparation of shape- and size-controlled AgNPs. Further investigations, including more extensive simulations of all colloid components (silver ions, citrate and tannic acid), are planned to gain a fuller understanding of the process.

## Electronic supplementary material

ESM 1 (DOCX 441 kb).
