# Supplementary material for: The role of tannic acid and sodium citrate in the synthesis of silver nanoparticles
Source: J Nanopart Res. 2017 Aug 4;19(8):273. doi: 10.1007/s11051-017-3973-9 (PMC5543188; doi:10.1007/s11051-017-3973-9)
Supplement: Supplementary file 1 — (DOCX 441 kb). [file 11051_2017_3973_MOESM1_ESM.docx]

**Supporting Information**

**The role of tannic acid and sodium citrate in the synthesis of silver nanoparticles**

Katarzyna Ranoszek-Soliwoda^1^, Emilia Tomaszewska^1^, Ewelina Socha^2^,
Pawel Krzyczmonik^2^, Anna Ignaczak^3^, Piotr Orlowski^4^, Małgorzata Krzyzowska^4^,

Grzegorz Celichowski^1^, Jaroslaw Grobelny^1^*

*^1^ Department of Materials Technology and Chemistry, Faculty of Chemistry,*

*University of Lodz, Pomorska 163, 90-236 Lodz, Poland*

*^2^ Department of Inorganic and Analytical Chemistry,Faculty of Chemistry,*

*University of Lodz, Tamka, 91-403 Lodz, Poland*

*^3^Department of Theoretical and Structural Chemistry, Faculty of Chemistry,
University of Lodz, Pomorska 163/165, 90-236 Lodz, Poland*

*^4^Department of Regenerative Medicine,
Military Institute of Hygiene and Epidemiology, Warsaw, Poland*

*Corresponding author e-mail address: j[grobel@uni.lodz.pl](mailto:grobel@uni.lodz.pl)(J. Grobelny)

Fax: +48 42 6355832

Tel. +48 42 6355837

***Measured and computed IR spectra of tannic acid – a comparison***

|  |
| --- |
|  |
|  |
|  |
|  |
|  |
| **Fig. 1S.** The IR spectra of tannic acid: experimentally measured (a) and calculated (using Lorentzian line shapes with the half-width 20) from the PM7 (b) and PM6 (c) results obtained for the most stable structures *in vacuo* (unscaled values). Note the different ranges for ν in the figures (a) and (b)–(c). |

**Table 1S.** Measured (ν_exp_) and calculated (ν_calc_) wavenumbers (in cm^–1^) of selected vibrations of tannic acid *in vacuo*, corresponding to the peaks marked in Figure 1S.

| No. | ν_exp_ | ν_calc PM7_ | ν_calc PM6_ | Description of main vibrations (PM7 results) |
| --- | --- | --- | --- | --- |
| 1 | 1710 | 1887, 1811 | 1854, 1757 | C=O stretching |
| 2 | 1612 | 1707 | 1638 | C_ar_–C_ar_ stretching |
| 3 | 1535 | 1607, 1579 | 1532, 1517 | C_ar_–C_ar_ stretching and C_ar_–OH stretching |
| 4 | 1446 | 1508 | 1449 | C_ar_–C_ar_ stretching and C_ar_–CO stretching |
| 5 | 1317 | 1371 | 1309 | C_ar_–OC stretching, C_ar_–O–H in-plane bending,  C_ar_–C_ar_ stretching |
| 6 | 1197 | 1296 | 1224 | O–CO and C_ar_–CO stretching, C_ar_–O–H in-plane bending, |
| 7 | 1087 | 1225 | 1132^a^ | C_glu_–C_glu_ stretching, C–H bending, C_ar_–C_ar_ stretching  (^a^ in PM6 this peak overlaps with peak no. 8 and the mode contains additionally C_ar_–C_ar_ and C–OH stretching) |
| 8 | 1029 | 1186 | 1097 | C–O–C asym. stretching |
| 9 | 958 | 1000,  965^b^ | 1015, 955 | C–O stretching, aromatic ring in-plane deformation (C_ar_–C_ar_–C_ar_ scissors), glucose deformation  ^b^ aromatic ring out-of-plane deformation (C_ar_–C_ar_–C_ar_ out-of-plane bending) |
| 10 | 872 | 882 | 845 | C_ar_–OH stretching, aromatic ring in-plane deformation (C_ar_–C_ar_–C_ar_ stretching) |
| 11 | 755 | (~770),  712 | (~755),  712 | O–C=O in-plane bending (scissors),  C_ar_–C_ar_(–C)–C_ar_ and O–C(–C)=O out-of-plane bending |

Although the shapes of experimental and computed spectra presented in Figure 1S differ significantly, all the most distinctive peaks observed in the measured data are reflected in the theoretical results. The differences in the positions of the peaks and the intensities may be due to the fact that the computed values are obtained for a molecule in vacuum, while the measurement is carried outunder entirely different conditions. Also, as mentioned in the main article, the theoretical part of our study is rather preliminary and based on the calculations done using semi-empirical methods, which are by nature approximate. More extended conformational searches and the use of more advanced theoretical methods would be desirable to verify the results presented.

***Method of calculations performed for tannic acid and its adducts with citric acid***

The initial models of all molecules studied were built in the program Hyperchem [1]. Three very different initial geometries of tannic acid were created by setting various, arbitrarily chosen orientations of the ‘branches’ attached to the glucose unit. The molecules were then optimized using the molecular mechanics method BIO+ (Charmm27) available in the program.

After initial optimization, for each of the three conformers of tannic acid the low-energy conformers in water were searched by performing simulations using the program Gabedit [2] combined with the MOPAC program [3]. The Stochastic Dynamics simulations (via Verlet algorithm) were performed at a temperature of 1000 K and with a total simulation time of 20 ps and a time step of 1 fs; for other parameters default values pre-defined in the Gabeditprogram were used. From the simulation trajectory the program selected the 50 lowest energy structures, thus from the three simulations 150 different conformers of tannic acid were obtained. In all these calculations the system was described by the semi-empirical method PM7. The solvent (water) was modelled by the conductor-like screening model COSMO [4]. The selected 150 structures were then optimized in water using the PM7 method, and among them the lowest energy structure of tannic acid was found (Figure 7 in the main article). An additional, shorter conformational search (setting a simulation time of 10 ps) was performed to obtain the most stable structure of citric acid in water.

The lowest energy conformers of tannic acid (TA) and citric acid (CA) were used to create the initial geometries of two structures of the TA–CA complex by placing the citric acid molecule in two different positions: in the first, the CA molecule was closer to one of the gallic units directly bound to glucose (‘internal unit’ denoted by the index ‘in’), while in the second it was closer to one of the gallic units more distant from glucose (‘external unit’ denoted by the index ‘ex’). From these two structures were created the corresponding complexes containing the oxidized forms of tannic acid (TA_ox_) by replacing the two COH groups (closest to CA) with C=O groups in either the internal or external gallic unit. For each of the four complexes a single simulation was performed, using the same parameters as those used in the simulations for tannic acid. Again the 50 lowest energy structures were selected and optimized in water at the PM7 theory level and the lowest energy structures presented in Figure 8 in the main article were found.

The interaction between citric acid and tannic acid was evaluated as a difference between the energies of the most stable complex found from the simulations and the energies obtained from the single point calculations performed for the structures of TA and CA extracted from the complex structure.

To obtain the IR spectra for tannic acid the lowest energy conformer found from thesimulations in water was re-optimized *in vacuo* using the PM7 and PM6 methods, and then for each of the resulting structures a vibrational analysis was performed, assuring that the spectrum obtained contains no imaginary frequencies. It should be mentioned that while the PM7 structure obtained after the re-optimization *in vacuo* is quite similar to that found in water, the PM6 structure *in vacuo* is significantly different (Figure 2S).


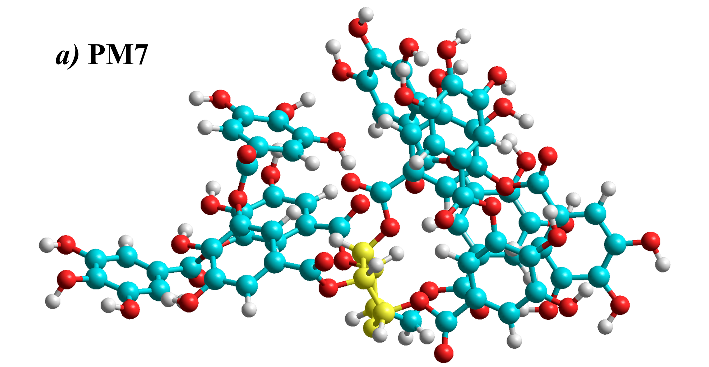

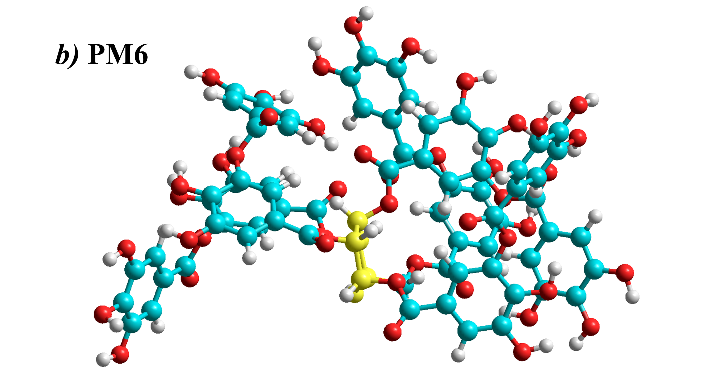


**Fig. 2S.** The structures obtained from the (a) PM7 and (b) PM6 optimizations performed *in vacuo*for the most stable structure found in water. Yellow denotes the heavy atoms that form the ring in the glucose unit.

***Literature***

1. HyperChem^(TM)^ Professional 8.0.10, Hypercube, Inc., 1115 NW 4th Street, Gainesville, Florida 32601, USA.
2. Gabedit 2.4.9, A. R. Allouche, J. Comput. Chem. 32 (2011) 174–182.
3. (a) MOPAC2016, Version: 16.299W. J. J. P. Stewart, Stewart Computational Chemistry, web: <http://OpenMOPAC.net>, (b) J. D. C. Maia, G. A. U. Carvalho, C. P. Mangueira, S. R. Santana, L. A. F. Cabral, G. B. Rocha, J. Chem. Theory Comput. 8 (2012) 3072–3081.
4. A. Klamtand G. Schüürmann. J. Chem. Soc. Perkin Transactions 2 (1993) 799–805.
